# Progress towards the United Nations 90‐90‐90 and 95‐95‐95 targets: the experience in British Columbia, Canada

**DOI:** 10.1002/jia2.25011

**Published:** 2017-11-13

**Authors:** Viviane D. Lima, Martin St‐Jean, Ignacio Rozada, Jean A. Shoveller, Bohdan Nosyk, Robert S. Hogg, Paul Sereda, Rolando Barrios, Julio S G Montaner

**Affiliations:** ^1^ British Columbia Centre for Excellence in HIV/AIDS St. Paul's Hospital Vancouver British Columbia Canada; ^2^ Division of AIDS Department of Medicine Faculty of Medicine University of British Columbia Vancouver British Columbia Canada; ^3^ School of Population and Public Health University of British Columbia Vancouver British Columbia Canada; ^4^ Faculty of Heath Sciences Simon Fraser University Burnaby British Columbia Canada; ^5^ Vancouver Community Health Services Vancouver Coastal Health Vancouver British Columbia Canada

**Keywords:** UNAIDS target, UN target, 90‐90‐90, 95‐95‐95, Diagnosis, Antiretroviral Therapy, Viral Suppression, Risk Factors, British Columbia

## Abstract

**Introduction:**

Antiretroviral therapy (ART) scale‐up is central to the global strategy to control the HIV/AIDS pandemic. To accelerate efforts towards ending the AIDS epidemic, the Joint United Nations Programme on HIV/AIDS released the 90‐90‐90 and 95‐95‐95 targets, which have recently been approved by the United Nations (UN). This study characterizes the province of British Columbia (BC)'s progress towards achieving the UN targets, predicts a trajectory up to 2030 according to each of the individual steps (i.e. %Diagnosed, %On ART and %Virologically Suppressed), and identifies the population sub‐groups at higher risk of not achieving these targets.

**Methods:**

The analyses were based on linked individual‐level datasets of people living with HIV (PLWH) in BC, aged ≥18 months, from 2000 to 2013. Using past trends in HIV prevalence and of each individual UN targets, we forecasted these outcomes until 2030 via generalized additive models. We ran a second set of analyses to assess the associations between individual demographic and behavioural factors and each of the individual steps of the UN targets. Lastly, we performed sensitivity analyses to account for uncertainty associated with prevalence estimates and suppression definitions.

**Results:**

Among the estimated 10666 PLWH in BC in 2013, 82% were diagnosed, 76% of those diagnosed were on ART and 83% of those on ART were virologically suppressed. We identified that females, PLWH aged <30 years and those with unknown risk or who self‐identify as having a history of injection drug use were the population subgroups that experienced the most challenge in engaging on ART and achieving viral suppression. Our model projections suggest that BC will achieve 90%‐91%‐90% and 97%‐99%‐97% by 2020 and 2030 respectively.

**Conclusions:**

As we approach 2020, BC is rapidly moving towards achieving the UN targets. However, region‐specific challenges persist. Identification of remaining regional challenges will be essential to achieving the proposed UN targets and therefore fulfilling the promise to end AIDS as a pandemic by 2030.

## 1 Introduction

Expanded access to antiretroviral therapy (ART) has a significant role to play in the global strategy to control the HIV/AIDS pandemic and alleviate its impact on people living with HIV (PLWH) 1. There is ample scientific evidence suggesting that early ART initiation not only reduces morbidity and mortality, but also reduces incidence rates of HIV, resulting in the concept of “Treatment as Prevention (TasP)” 2. Definitive evidence of the effect of ART on HIV transmission via heterosexual contact was provided by the HTPN 052 randomized control trial 3. This finding was verified among men who have sex with men in the PARTNER study 4, and further supported by a number of observational and modelling population‐based studies 5, 6, 7.

In order to achieve maximum benefits from the expansion of TasP, it is crucial that PLWH are diagnosed early in the course of their HIV infection, linked and retained in HIV care, offered treatment at diagnosis, and highly adherent to attain and sustain full virologic suppression 8. As a result, monitoring engagement in the “HIV cascade of care” is now widely recognized as a critical element of HIV surveillance, aimed at ensuring PLWH can fully benefit from existing management strategies, including immediate access to ART to prevent disease progression to AIDS and premature death, and to minimize the risk of ongoing HIV transmission.

Controlling the global HIV pandemic will require renewed political will 2. At the 2014 International AIDS Conference in Melbourne, the Joint United Nations Programme on HIV/AIDS proposed the 90‐90‐90 and 95‐95‐95 targets to accelerate efforts towards ending the AIDS epidemic by 2030, which have recently been approved by the United Nations (UN) 9, 10. The 90‐90‐90 and 95‐95‐95 targets proposes that by 2020 and 2030, respectively, at least 90% and 95% of all PLWH should be diagnosed, at least 90% and 95% of those diagnosed should be on ART and at least 90% and 95% of those on ART should be virologically suppressed, translating into a final target of at least 73% and 86% of all PLWH achieving virologic suppression. UN Modelling has suggested that the full implementation of the 90‐90‐90 target could lead to an approximate 90% decrease in AIDS‐related deaths and an approximate 90% decrease in new HIV infections by 2020, compared with the status quo using 2010 as the baseline 9.

In 2003, the province of British Columbia (BC), Canada, initiated and progressively scaled up efforts to expand HIV testing and publicly funded ART access, as part of a province‐wide initiative to decrease HIV‐related morbidity and mortality as well as HIV transmission 11, 12. This study characterizes BC's progress towards achieving the 90‐90‐90 and 95‐95‐95 targets, predicts a trajectory up to 2030 according to each of the individual steps (i.e. %Diagnosed, %On ART and %Suppressed), and identifies the population sub‐groups at higher risk of not achieving these targets.

## 2 Methods

### 2.1 Data sources

Eligible study participants were recruited from the BC Seek and Treat for Optimal Prevention of HIV/AIDS population‐based cohort (STOP HIV/AIDS), which contains longitudinal individual‐level data on all PLWH ever diagnosed in the province. This population‐based cohort is derived from various linkages among provincial databases (see Data S1) 11, 13, 14, 15, 16, 17, 18. The BC Centre for Excellence in HIV/AIDS (BC‐CfE) is responsible for distributing ART, at no cost and without co‐payments, to all PLWH in the province, and its database captures HIV plasma viral load (pVL) results for all its participants. Previous studies have described these databases and the respective linkages 11, 19. Annual HIV prevalence estimates for BC from 2000 to 2013 were derived from the Public Health Agency of Canada (PHAC) available estimates of the percent of PLWH who were unaware of their infection, and the number of individuals diagnosed based on the STOP HIV/AIDS cohort 20, 21, 22. For more detail, please see Figure S1.

### 2.2 Outcomes and statistical analysis

The inclusion criteria consisted of all diagnosed PLWH enrolled in the STOP HIV/AIDS cohort, aged ≥18 months old, in each calendar year from 2000 to 2013 in BC. The definition for each of the steps of the 90‐90‐90 or 95‐95‐95 targets, for each calendar year, is as follows:

- %Diagnosed (among all PLWH in BC), the first instance of one of the following: a confirmed HIV‐positive test, a detectable pVL >50 copies/ml, an HIV‐related physician visit or hospitalization, a reported AIDS‐defining illness or the dispensation of antiretroviral treatment during the calendar year. This definition has been previously validated 11.
- %On ART (among PLWH who have been diagnosed): receiving antiretroviral treatment at least three months apart within the calendar year 11.
- %Suppressed (among PLWH on ART): In our setting, multiple pVL tests are typically available per patient within a calendar year (median number of pVLs per year per patient was 4 (25th to 75th percentile three to five or six depending on the year)). As such, we used a conservative definition that utilizes all available pVLs from each patient, i.e. those who had an undetectable pVL test (<200 copies/ml) at all times tested in a given calendar year.

We modelled the non‐linear trend in HIV prevalence and each of the individual steps of the UN targets via generalized additive models 23, 24, 25. When modelling the HIV prevalence, we assumed a negative binomial distribution and the log link function, and for the proportions for each of the individual targets, we assumed a beta distribution and the cloglog link function (chosen after examining the data and goodness of fit statistics). In brief, we used a cubic regression spline to smooth the trend in these outcomes in which calendar year was the explanatory variable being smoothed. Additionally, different models were fitted based on the number of knots used to smooth the trend in all outcomes when the estimation was via restricted maximum likelihood. We also used a built‐in smoothing parameter estimation method “GCV.Cp” to estimate the number of knots in each of the models. To assess the goodness of fit of each model, R outputs the adjusted R^2^, the percentage of the deviance explained, a test to check the appropriateness of the number of knots in the model, and the sum of the deviance residuals. Subsequently, we forecasted the evolution of the HIV prevalence and each target from 2014 to 2030. In these analyses, we used the statistical software R© version 3.2.2 mgcv library (functions gam, summary, gam.check, residuals.gam and forecast). All *p*‐values were two‐sided and the significance level was set at 5%.

We ran a second set of analyses to assess the associations between individual demographic and behavioural factors and each of the individual UN targets. The binary outcome was classified as yes (i.e. successful in achieving a particular target) vs. no (i.e. failing to achieve a particular target). The possible explanatory variables, measured at diagnosis, were calendar year (continuous), age (<18, 18 to 29, 30 to 39, 40 to 49 and ≥50 years), gender (male/female) and HIV risk group (gay, bisexual and other men who have sex with men (MSM); history of injection drug use (IDU); MSM/IDU; other; unknown). Multivariable explanatory logistic regression models were built to assess which of these factors were associated with each of the individual steps of the UN targets. A modified backward stepwise technique developed and validated by our group, based on the Akaike Information Criterion and Type III *p*‐values, was used in the selection of explanatory variables 26. Categorical variables were compared using the Cochran–Mantel–Haenszel test, proportions were compared using a two proportion z‐score test, and trends in counts were tested using a Poisson regression 27, 28. These analyses were performed using R© 3.2.2 MASS library and SAS, version 9.4 (SAS, Cary North CA, USA).

### 2.3 Sensitivity analyses

There are several potential sources of uncertainty associated with HIV prevalence estimates. Thus, we performed a sensitivity analysis considering different scenarios of prevalence estimates. Since the percentages of the “on ART” and “virologically suppressed” steps of the UN targets remained unchanged irrespective of the prevalence estimates, we calculated the percentage of diagnosed PLWH for three scenarios of prevalence estimates utilizing the original PHAC estimates, and by decreasing these prevalence estimates by 2% and 5%. The second sensitivity analysis focused on the definition of viral suppression. In this case, we ran these analyses utilizing PHAC's definition, which is less conservative, and defines the %Suppressed (among PLWH on ART) as patients whose latest pVL test was undetectable (<200 copies/ml) in the calendar year.

### 2.4 Ethical considerations

The University of BC Ethics Review Committee at the St. Paul Hospital site provided ethics approval for this study (H08‐02095; H05‐50123).

## 3 Results

### 3.1 Historical trends

From 2000 to 2013, HIV prevalence (diagnosed and undiagnosed) in BC increased from 9150 to 10666 cases (17% increase; *p*<0.0001). In this same period, the number of diagnosed PLWH increased from 5954 to 8736 (47% increase; *p*<0.0001), the number on ART increased from 2807 to 6605 (135% increase; *p*<0.0001) and the number of those virologically suppressed increased from 1287 to 5459 (324% increase; *p*<0.0001, Table S1). Thus, in BC, between 2000 and 2013, the %Diagnosed increased from 65% to 82% (26% increase; *p*<0.0001), the %On ART (among diagnosed PLWH) increased from 47% to 76% (60% increase; *p*<0.0001), and the %Suppressed (among those on ART) increased from 46% to 83% (80% increase; *p*<0.0001); altogether this translates into 14% of all PLWH virologically suppressed in 2000 and 51% in 2013 (264% increase; *p*<0.0001).

### 3.2 Characteristics of PLWH ever diagnosed with HIV

Since 2000, there was a total of 12976 PLWH ever diagnosed with HIV in the province. The majority of these individuals were male (80%), aged 30 to 49 years (64%) and self‐identified either as a MSM (29%) or as an IDU (27%, Table 1). Table 2 present demographic and behavioural factors associated with each of the individual steps of the UN targets, excluding diagnosed, given that all STOP HIV/AIDS participants have been diagnosed in compliance with the cohort entry criteria. We observed that, in comparison to females, males were 43% more likely to be on ART and 26% more likely to achieve virologic suppression. In terms of age, in comparison to PLWH aged 30 to 39 years, individuals <18 and 18 to 29 years were less likely to be on ART and to achieve virologic suppression, whereas those ≥40 years performed better in both outcomes. Regarding the HIV risk group, in comparison to MSM, all exposure groups were less likely to be on ART (IDUs 49%, other risk 21% and unknown risk 82%). For virologic suppression, all risk groups were less likely to succeed than MSM (IDUs 57% and remaining groups ranged from 5% to 57%). Individuals diagnosed more recently were more likely to be on ART and to achieve virologic suppression as compared to the previous year (odds ratio 1.15 and 1.16 respectively).

**Table 1:** Descriptive statistics of the 12976 individuals in British Columbia ever diagnosed with HIV from 2000 to 2013

Variables	Ever diagnosed	On ART		*p*‐value	Suppressed (percent of all viral loads <200 copies/ml)		*p*‐value	Suppressed (last viral load <200 copies/ml)		*p*‐value
		Yes	No		100%	<100%		Yes	No	
	(N=12976)	(N=8970)	(N=4006)		(N=7590)	(N=1355)		(N=8416)	(N=529)	
Gender				<0.0001			<0.0001			<0.0001
Male	10319 (80%)	7383 (72%)	2936 (28%)		6367 (86%)	995 (14%)		6984 (95%)	378 (5%)	
Female	2604 (20%)	1555 (60%)	1049 (40%)		1209 (78%)	342 (22%)		1403 (90%)	148 (10%)	
Unknown	53 (0%)	32 (60%)	21 (40%)		14 (44%)	18 (56%)		29 (91%)	3 (9%)	
Age (years)				0.0004			<0.0001			0.0003
<18										
18 to 29	167 (1%)	92 (55%)	75 (45%)		77 (84%)	15 (16%)		86 (93%)	6 (7%)	
30 to 39	2461 (19%)	1640 (67%)	821 (33%)		1330 (81%)	306 (19%)		1520 (93%)	116 (7%)	
40 to 49	4842 (37%)	3505 (72%)	1337 (28%)		2952 (84%)	546 (16%)		3268 (93%)	230 (7%)	
≥50	3542 (27%)	2532 (71%)	1010 (29%)		2184 (87%)	338 (13%)		2404 (95%)	118 (5%)	
HIV risk group				<0.0001			<0.0001			<0.0001
MSM	3755 (29%)	3216 (86%)	539 (14%)		2893 (90%)	321 (10%)		3111 (97%)	103 (3%)	
IDU	3501 (27%)	2530 (72%)	971 (28%)		1963 (78%)	561 (22%)		2263 (90%)	261 (10%)	
MSM/IDU	933 (7%)	840 (90%)	93 (10%)		719 (86%)	118 (14%)		795 (95%)	42 (5%)	
Other	1529 (12%)	1180 (77%)	349 (23%)		996 (85%)	178 (15%)		1113 (95%)	61 (5%)	
Unknown	3258 (25%)	1204 (37%)	2054 (63%)		1019 (85%)	177 (15%)		1134 (95%)	62 (5%)	

MSM, gay, bisexual and other men who have sex with men; IDU, history of injection drug use; *p* for gender did not include the unknown category, and the actual percentage for the unknown category is 0.4%.

**Table 2:** Multivariable models utilizing the data of the 12976 individuals in British Columbia ever diagnosed with HIV from 2000 to 2013

Variables	Adjusted odds ratio (95% confidence interval)		
	On ART	Suppressed (having an undetectable viral load (<200 copies/ml) at all tests)	Suppressed (last viral load <200 copies/ml)
Gender			
Female	1 (–)	1 (–)	1 (–)
Male	1.43 (1.38 to 1.49)	1.26 (1.19 to 1.33)	1.21 (1.12 to 1.31)
Age (years)			
<18	0.82 (0.73 to 0.92)	0.53 (0.45 to 0.63)	0.36 (0.30 to 0.45)
18 to 29	0.64 (0.61 to 0.66)	0.78 (0.74 to 0.83)	0.79 (0.74 to 0.85)
30 to 39	1 (–)	1 (–)	1 (–)
40 to 49	1.14 (1.11 to 1.18)	1.27 (1.21 to 1.33)	1.50 (1.41 to 1.61)
≥50	1.13 (1.08 to 1.18)	1.41 (1.32 to 1.51)	1.82 (1.65 to 2.01)
HIV risk group			
MSM	1 (–)	1 (–)	1 (–)
IDU	0.51 (0.49 to 0.53)	0.43 (0.41 to 0.46)	0.37 (0.35 to 0.40)
MSM/IDU	0.93 (0.89 to 0.98)	0.56 (0.53 to 0.60)	0.51 (0.46 to 0.55)
Other	0.79 (0.75 to 0.83)	0.86 (0.80 to 0.92)	0.87 (0.79 to 0.96)
Unknown	0.18 (0.17 to 0.18)	0.95 (0.89 to 1.02)	0.96 (0.87 to 1.07)
Calendar year	1.15 (1.14 to 1.15)	1.16 (1.16 to 1.17)	1.22 (1.21 to 1.23)

MSM, gay, bisexual and other men who have sex with men; IDU, history of injection drug use. All models included gender, age, HIV risk group and calendar year.

**Figure 1:**
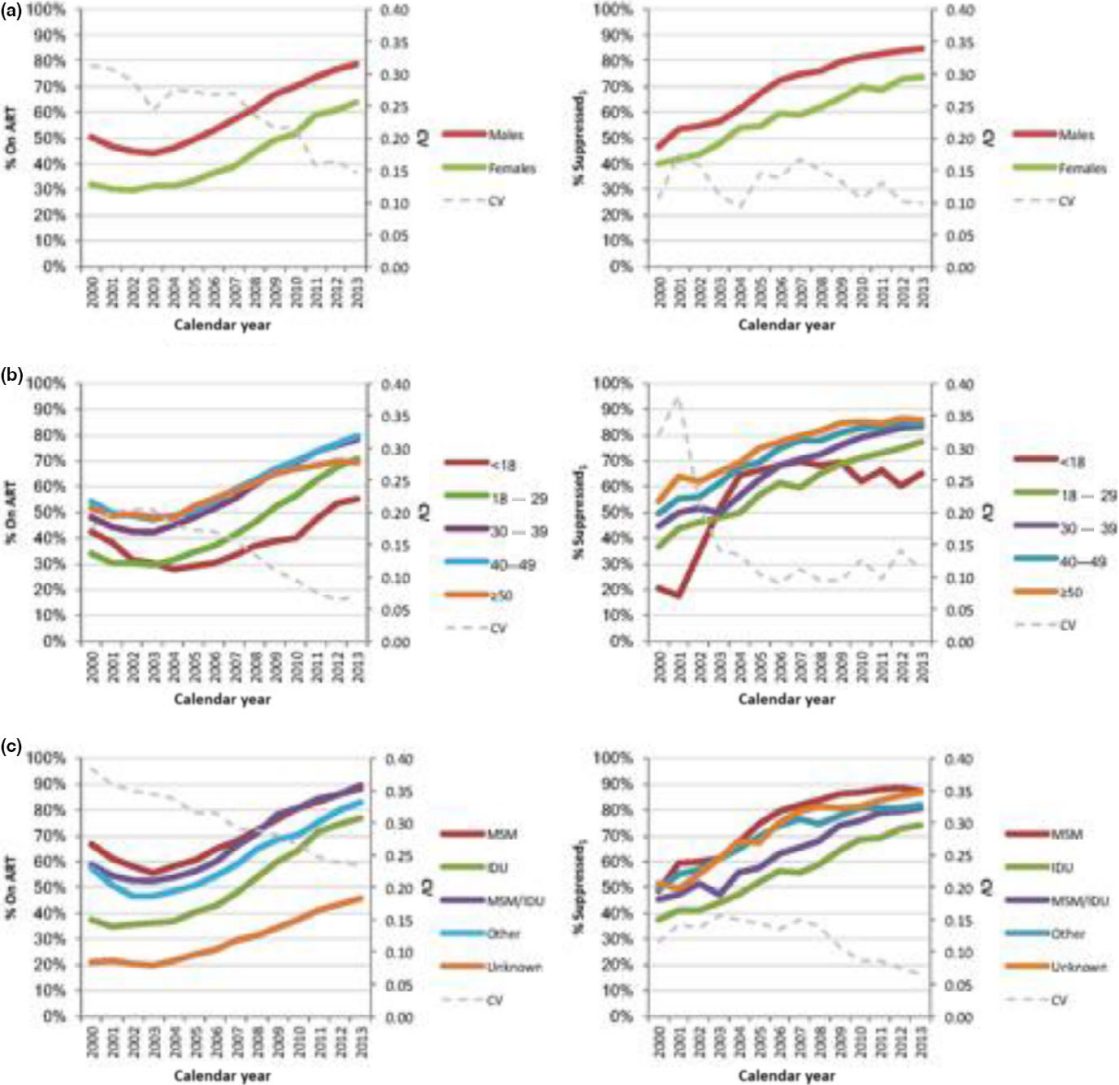
UN targets trajectory for British Columbia, from 2000 to 2013, stratified by: (A) gender, (B) age group and (C) HIV risk group. Suppressed_1_, having an undetectable viral load (<200 copies/ml) at all tests per calendar year; MSM, gay, bisexual and other men who have sex with men; IDU, history of injection drug use; CV, coefficient of variation; UN, United Nations.

Over time, we observed that the gap in the %On ART and %Suppressed by gender, age group and HIV risk group has decreased (see trend in the coefficient of variation in Figure 1). In Figure 1, we identified that females, PLWH aged <30 years and those with unknown risk or who self‐identify as IDU were the population subgroups that experienced the most challenge in engaging on ART. These same sub‐groups, except for those with unknown risk, had the most difficulty in achieving viral suppression.

### 3.3 Projections

**Figure 2:**
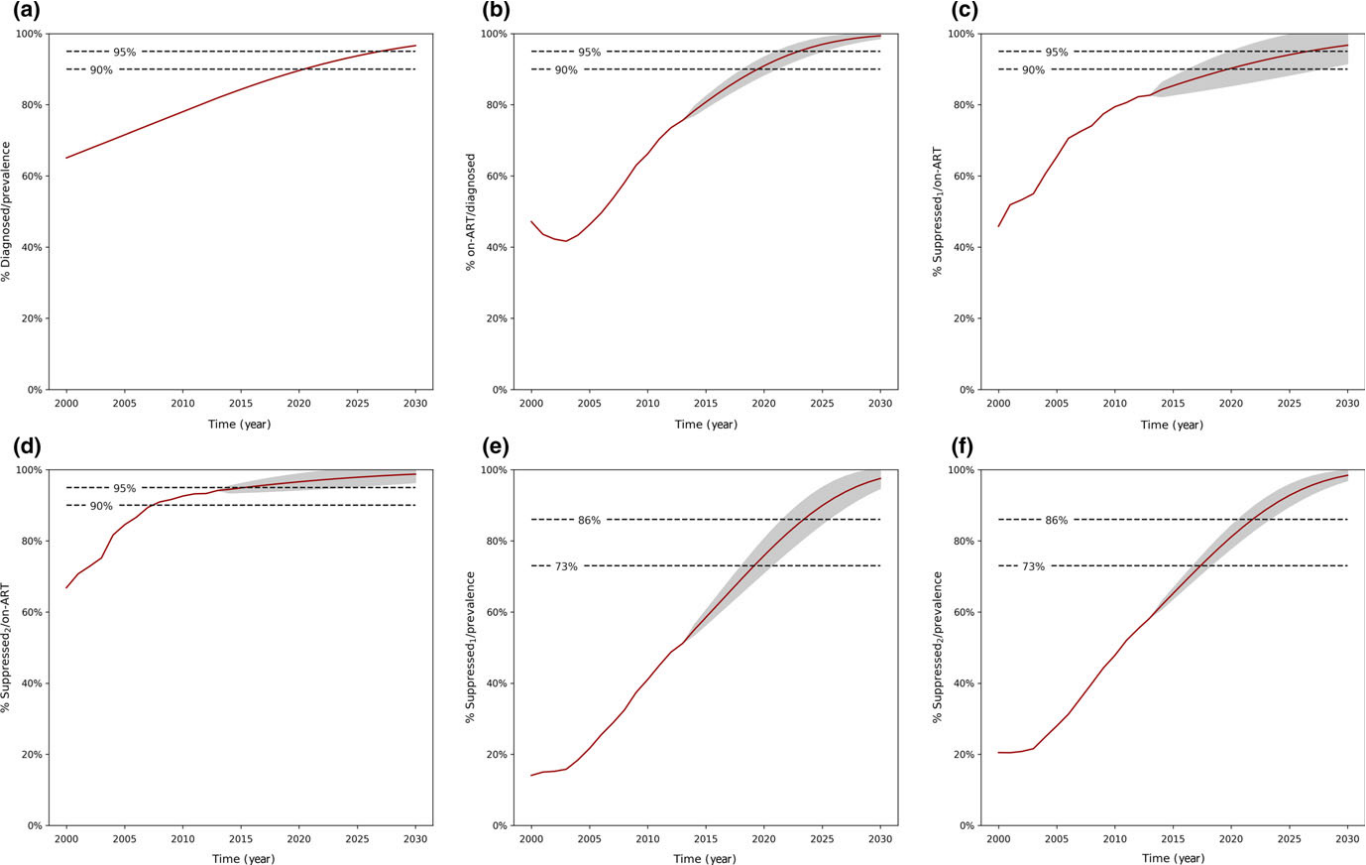
UN targets trajectory for British Columbia, by calendar year, from 2000 to 2030. Prevalence: Adjusted PHAC estimates; Suppressed_1,_ having an undetectable viral load (<200 copies/ml) at all tests; Suppressed_2_, last viral load <200 copies/ml; UN, United Nations. Area in grey describes the 95% Confidence Interval around the estimates. See Tables S2, S3, S4 for model fit, and forecast estimates.

Our model projections suggest that in BC, in 2014 the HIV prevalence was 10673 (95% Confidence Interval 10433 to 10912), 83% (83% to 83%) were diagnosed, 78% (77% to 80%) were on ART (among diagnosed) and 84% (82% to 86%) were suppressed (among those on ART). By 2020 the HIV prevalence will be 10639 (9885 to 11393) and by 2030 it will be 10584 (8959 to 12209) cases. We also estimated that by 2020, 90% (90% to 90%) of PLWH will be diagnosed, 91% (88% to 93%) will be on ART and 90% (85% to 95%) will be suppressed. In 2030, these percentages will be 97% (97% to 97%), 99% (99% to 100%) and 97 (92% to 100%) respectively (Table 3). The ultimate target for the 90‐90‐90 of at least 73% of all PLWH being virologically suppressed will be reached in 2020, and of 86% for the 95‐95‐95, will be reached in 2024 (Figure 2). For more detail on these projections, please see Tables S2, S3, S4.

**Table 3:** Model projections for the UN targets at the end of 2014, 2020 and 2030, in British Columbia

Year	Prevalence estimates	PHAC prevalence estimates	2% Decrease in PHAC prevalence estimates	5% Decrease in PHAC prevalence estimates
A) Prevalence estimate scenarios (95% confidence interval)				
2014	10673 (10433 to 10912)	11758 (11496 to 12019)	11489 (11221 to 11757)	11140 (10877 to 11404)
2020	10639 (9885 to 11393)	12011 (11121 to 12901)	11639 (10713 to 12564)	11295 (10388 to 12202)
2030	10584 (8959 to 12209)	12445 (10419 to 14472)	11892 (9806 to 13978)	11557 (9511 to 13602)

Year	Diagnosed/prevalence	Diagnosed/prevalence_1_	Diagnosed/prevalence_2_	Diagnosed/prevalence_3_
B) Percent diagnosed by prevalence estimate scenario (95% confidence interval)				
2014	83% (83% to 83%)	76% (75% to 76%)	77% (77% to 78%)	80% (79% to 80%)
2020	90% (90% to 90%)	82% (80% to 83%)	83% (81% to 85%)	86% (84% to 87%)
2030	97% (97% to 97%)	90% (87% to 92%)	91% (88% to 94%)	93% (91% to 96%)

Year	On ART/diagnosed	Suppressed_1_/on ART	Suppressed_2_/on ART	Suppressed_1_/prevalence	Suppressed_2_/prevalence
C) Steps of the UN target (95% confidence interval)					
2014	78% (77% to 80%)	84% (82% to 86%)	94% (93% to 95%)	55% (54% to 56%)	62% (61% to 63%)
2020	91% (88% to 93%)	90% (85% to 95%)	97% (94% to 99%)	76% (71% to 81%)	81% (78% to 84%)
2030	99% (99% to 100%)	97% (92% to 100%)	99% (96% to 100%)	98% (95% to 100%)	98% (97% to 100%)

Prevalence, adjusted PHAC estimates; Prevalence_1_, Public Health Agency of Canada (PHAC) prevalence estimates; Prevalence_2_, 2% decrease in PHAC prevalence estimates; Prevalence_3_, 5% Decrease in PHAC prevalence estimates; Suppressed_1_, having an undetectable viral load (<200 copies/ml) at all tests; Suppressed_2_, last viral load <200 copies/ml; UN, United Nations. See Tables S2, S3, S4 for model fit and forecast estimates.

### 3.4 Sensitivity analyses

No scenario explored in our prevalence estimate sensitivity analysis met the 90% and 95% targets for the proportion diagnosed by 2020 and 2030 respectively (Table 3). The proportion diagnosed varied between 82% to 86% and 90% to 93% respectively. It should be noted that these forecasted scenarios with higher prevalence estimates demonstrate that when the undiagnosed proportion of the prevalence increases relative to that of the original prevalence estimates, incident HIV infections associated with undiagnosed transmission will cause prevalent HIV cases to rise steadily from 2014 to 2020, and from 2020 to 2030.

When focusing on the operational definition of virologic suppression, we estimated the %Suppressed as the last pVL being <200 copies/ml (i.e. PHAC's definition). In this case, the data showed that we have already reached the 90% target in 2008 and the 95% target will be reached in 2015 for %Suppressed among those on ART. For the ultimate target of 73% and 86% for %Suppressed among all PLWH, we estimated that they will be reached in 2018 and 2022 respectively.

## 4 Discussion

This represents the first study to characterize past, current and future progress towards the UN 90‐90‐90 and 95‐95‐95 targets in BC by 2020 and 2030 respectively. Our model projections suggest BC is on track to achieve both targets. Further analyses demonstrated that females, younger individuals, IDUs and those with unknown HIV risk group were the population sub‐groups experiencing the most challenges in achieving these goals.

The observed gender differences in ART uptake and virologic suppression demonstrated in our study are not unprecedented 29, 30, 31. However, in both cases, the overall evidence is conflicting 32, 33, 34. The gender differences found in different studies may be largely related to the heterogeneity in demographic factors (e.g. geography, income, ethnicity), psychosocial (e.g. substance use, sexual behaviour) and treatment‐related factors (e.g. treatment adherence) pertaining to this population. The observed association between history of IDU and lower ART uptake and virologic suppression is also long‐standing 35, 36. Individuals with a history of IDU may find themselves in situations where they struggle to optimally manage their health, which can be further complicated by active addictions and socio‐structural, individual‐level and provider‐based barriers to care 35, 37. Thus, to optimize our ability to meet the UN targets, it is critical that further efforts be deployed towards optimizing engagement of specific sub‐populations, as identified in our study.

At the end of 2016, the PHAC released the progress towards the UN 90‐90‐90 target for Canada for the year 2014 38. It estimated that in 2014, 80% of PLWH were diagnosed, 76% were on ART (among diagnosed PLWH) and 89% were suppressed (among PLWH on ART) in Canada. These compare unfavourably against BC if we use the same definition for virologic suppression, as we estimated that in 2014, in BC, 83% were diagnosed, 78% on ART and 94% were suppressed. BC's current and projected success is better interpreted with a brief acknowledgement of important contextual information. BC is the only jurisdiction in Canada that provides universally covered, fully subsidized ART as well as laboratory and medical monitoring services. At the core of BC's approach to the expansion of TasP were five guiding principles which included: combating stigma and discrimination, reach and engagement, community participation, aboriginal involvement and simplified consent for testing and engagement into care 39. Since 2003, TasP has been scaled‐up progressively and evaluated extensively in BC 11, culminating with the implementation of the province‐wide seek/test/treat strategy in 2010 40. Key aims included the normalization of HIV testing in the general population, supports to facilitate access to free ART, and extensive deployment of harm reduction strategies 11, 41, 42. These efforts, in addition to improvements in compliance with HIV care guidelines and clinical response to treatment, have resulted in greater engagement of PLWH in the HIV cascade of care and overall reduced HIV transmission 11.

Our sensitivity analysis examining scenarios bring forward the challenge regarding the long‐term feasibility and sustainability of the HIV care and treatment in BC in the context of increasing prevalence. It should be noted that in BC, we have shown that the expansion of ART is highly cost‐effective even in a scenario of increased prevalence, largely due to substantial decreases in morbidity and mortality 43. However, an increase in prevalent cases will translate into an increase in resource utilization in the province. With the ongoing commitment of the Provincial Government and the rapidly available generic formulations of the most utilized regimen combinations, we are confident that PLWH in BC will continue to receive appropriate care.

In light of the global consensus on the UN targets 10, the findings of this study should help inform the continued scale‐up of TasP strategies in other contexts 44, 45, 46, 47, 48, 49, 50. In the United States, 86% of PLWH were diagnosed, but only 42% of them were on ART, and 82% of those who were on ART were virologically suppressed in 2011 [44.] The majority (70%) of the countries in Europe that reported data on the percentage of diagnosed PLWH on ART were under 70%. Conversely, nearly two‐thirds (65%) of the countries that reported data for the percentage of diagnosed PLWH were between 70% to 89% 45. Only Sweden reported having met the UN 90‐90‐90 target 49. The need for expanded testing is particularly evident in Sub‐Saharan Africa, as well as much of Asia and the Pacific region. In sub‐Saharan Africa, only 45% of PLWH were diagnosed, whereas 86% of the diagnosed were on ART, and 76% of those on ART were virologically suppressed in 2013 9. Encouragingly, a population‐based random sample across 30 rural and peri‐urban communities in Botswana found that 83.3% of PLWH were diagnosed, 87.4% of them were on ART and 96.5% of those who were on ART were virologically suppressed from October 30, 2013 to November 24, 2015 51. The recent success in Botswana underlines the feasibility of the UN 90‐90‐90 target in resource‐limited settings, where the HIV burden is the highest 46. In Asia and the Pacific region, many countries also experienced difficulty in diagnosing PLWH (≤35%) such as Indonesia and Bangladesh and Pakistan 48.

When comparing the performance between settings, we should note that we cannot establish with absolute certainty that the differences observed reflect a difference in testing and treatment coverage, quality of and access to care, regimen adherence and treatment options as opposed to a variation in the definitions or data sources and quality used to obtain estimates. However, individual, community and systemic barriers continue to negatively affect engagement of PLWH along each stage of the cascade. For example research has identified financial constraints as a significant barrier to ART adherence in resource‐rich settings such as in the United States, where co‐payments are higher 52. Stigma also remains a crucial barrier, both perceived and actual stigma have been linked to reduced levels of HIV testing, retention in care and ART adherence in a variety of settings 53, 54. In resource‐limited settings, capacity for viral load testing persists as a barrier with regards to monitoring progress. Many countries are not in a position to provide all PLWH on ART with viral load testing. Nonetheless, to meet and appropriately monitor the UN targets, all PLWH who initiate therapy will require access to viral load testing 9.

There are some limitations in our study. First, the actual prevalence of HIV cases from 2000 to 2013 is not definitively known and was thus derived from PHAC's modelling estimates which also provided an independent source for the number of PLWH who were unaware of their infection 20. However, our sensitivity analysis examined different prevalence scenarios to account for the uncertainty associated with our prevalence estimates. Regarding demographic variables, we were unable to account for the role of ethnicity in achieving each step of the targets due to minimal data availability. Furthermore, while the definition for %Diagnosed used in this paper has been validated empirically 11, our definition differs from the one ascribed by the BC Centre for Disease Control in order to comprehensively capture all HIV cases in the province. Note that HIV cases identified through BC Centre for Disease Control accounts for 91% of the diagnosed individuals included in this study. Lastly, as in any model projection, caution is warranted regarding our projected numbers, especially for 2030, since they were based on historical trends. As such, important changes in policies, social and economic factors may influence these projections.

## 5 Conclusions

In summary, BC (Canada) is in a position to reach the UN targets by 2020 and 2030. However, there are potential challenges in achieving these targets which should be further addressed in order to further reduce HIV morbidity, mortality and HIV transmission, thereby creating favourable conditions to meet the goal of “ending the AIDS pandemic by 2030.” Thus, despite of these challenges, our results provide strong evidence that integrated comprehensive free programs that facilitate testing and the delivery of treatment and care to PLWH can be effective in controlling, and eventually ending the AIDS pandemic.

## Competing interests

We have the following conflicts of interest: Montaner has received limited unrestricted funding, paid to his institution, from Abbvie, Bristol‐Myers Squibb, Gilead Sciences, Janssen, Merck, and ViiV Healthcare. The remaining authors do not have conflicts to declare.

## Authors’ contributions

Lima and Montaner involved in study concept and design. Lima, Sereda and Montaner involved in acquisition of data. Lima and St‐Jean performed drafting of the manuscript. Lima, St‐Jean, Shoveller, Nosyk, Hogg, Montaner, Rozada, Sereda and Barrios contributed to critical revision of the manuscript for important intellectual content and for final approval. Lima also performed analysis and interpretation of data, statistical analysis, obtained funding and administrative, technical or material support of the manuscript. Montaner involved in study supervision .

## Funding

Montaner is supported with grants paid to his institution by the British Columbia Ministry of Health and by the US National Institutes of Health (R01DA036307). Lima is funded by two grants from the Canadian Institutes of Health Research (MOP‐125948 and PJT‐148595), by a Scholar Award from the Michael Smith Foundation for Health Research and a New Investigator award from the Canadian Institutes of Health Research.

## Disclaimer

The sponsors had no role in the design, data collection, data analysis, data interpretation, or writing of the report. The corresponding author had full access to all data in the study and had final responsibility to submit for publication.

## Supporting information


**Data S1.** Description of the databases in the STOP HIV/AIDS cohort.
**Table S1.** UN targets trajectory for British Columbia (all individuals aged ≥18 months), by calendar year, from 2000 to 2013.
**Table S2.** Diagnostic statistics and model coefficients for the generalized additive model for each outcome among all 12976 individuals (aged ≥18 months). (A) Negative binomial distribution and log link function. (B) Beta distribution and the cloglog link function.
**Table S3.** UN targets trajectory for British Columbia (all individuals aged ≥18 months), by calendar year, from 2000 to 2030.
**Table S4.** UN targets trajectory for British Columbia (all individuals aged ≥18 months), by calendar year, from 2000 to 2013.
**Figure S1.** Methodology for adjusted prevalence estimates.
