# Supplementary material for: Progress towards the United Nations 90‐90‐90 and 95‐95‐95 targets: the experience in British Columbia, Canada
Source: J Int AIDS Soc. 2017 Nov 13;20(3):e25011. doi: 10.1002/jia2.25011 (PMC5810311; doi:10.1002/jia2.25011)

**Progress Towards the United Nations 90-90-90 and 95-95-95 Targets: The Experience in British Columbia, Canada**

§ Corresponding author: Viviane D. Lima (vlima@cfenet.ubc.ca)

This study was carried out using data from the British Columbia Seek and Treat for Optimal Prevention of HIV/AIDS (STOP HIV/AIDS) population-based cohort, which is derived from various linkages between provincial administrative databases.

**Data Steward: British Columbia Centre for Disease Contro**l(1, 2)

i. Provincial HIV/AIDS Surveillance Database: a surveillance database that collates all HIV testing, new HIV diagnosis and occurrence of AIDS-defining illnesses data;

**Data Steward: British Columbia Centre for Excellence in HIV/AIDS**(3)

ii. Drug Treatment and Laboratory Databases, which captures all antiretroviral dispensing data, plasma viral load testing, drug resistance testing, occurrence of AIDS-defining illnesses, approximately 85% of CD4 cell count measurements, and key patient demographic information;

**Data Steward: British Columbia Ministry of Health**(4)

iii. The Medical Services Plan (MSP) billing database, which captures HIV and non-HIV-related inpatient and outpatient services provided by physicians and supplementary health care practitioners, as well as diagnostic procedures. This database also contains cost associated with claims paid through fee-for-service and the Alternative Payment Program;

iv. Home and Community Care database, which captures a variety of services including hospice and home nursing care, adult day services, assisted living, respite care, residential and convalescent care

v. Mental Health Services database, which captures utilization of mental health services including fee-for-service, institutional care, community clinics and acute care.

vi. The PharmaNet database is a real-time system, which captures all prescriptions for drugs and medical supplies dispensed from community pharmacies in BC as well as prescriptions dispensed from hospital outpatient pharmacies use at home. Note that this database does not capture antiretroviral dispensing data;

vii. The Client Roster or Consolidation File, which captures individual demographic and geographic data. This database is also used to construct population denominators;

**Data Steward: Canadian Institute for Health Information**(5)

viii. The Discharge Abstract Database (DAD), which captures all discharges, transfers and deaths of in-patients and day surgery patients from acute care hospitals across BC;

**Data Steward: British Columbia Vital Statistics Agency**(6)

ix. The Vital Statistics database, which records death information of all BC’s residents.

**References**

1. British Columbia Centre for Disease Control (2016):. HIV/AIDS Information System (HAISYS). Clinical Prevention Services, British Columbia Centre for Disease Control. Data Extract. MOH (2016). [Available from: <http://www.bccdc.ca/about/accountability/data-access-requests/public-health-data>.

2. British Columbia Centre for Disease Control Public Health Laboratory (2016):. HIV laboratory testing datasets (tests: ELISA, Western blot, NAAT, p24, culture). Clinical Prevention Services, British Columbia Centre for Disease Control. MOH (2016) [Available from: <http://www.bccdc.ca/about/accountability/data-access-requests/public-health-data>.

3. Patterson S, Cescon A, Samji H, Cui Z, Yip B, Lepik KJ, et al. Cohort Profile: HAART Observational Medical Evaluation and Research (HOMER) cohort. Int J Epidemiol. 2015;44(1):58-67.

4. British Columbia Ministry of Health (2016):. Medical Services Plan (MSP) Payment Information File; Consolidation File (MSP Registration & Premium Billing); Home & Community Care (Continuing Care); Mental Health; PharmaNet. British Columbia Ministry of Health. Data Extract. MOH (2016) [Available from: <http://www2.gov.bc.ca/gov/content/health/conducting-health-research-evaluation/data-access-health-data-central>.

5. Canadian Institute of Health Information (2016):. Discharge Abstract Database (Hospital Separations). British Columbia Ministry of Health. Data Extract. MOH (2016) [Available from: <http://www2.gov.bc.ca/gov/content/health/conducting-health-research-evaluation/data-access-health-data-central>.

6. British Columbia Vital Statistics Agency (2016):. Vital Statistics Deaths. British Columbia Ministry of Health. Data Extract. MOH (2016) [Available from: <http://www2.gov.bc.ca/gov/content/health/conducting-health-research-evaluation/data-access-health-data-central>

**Supplementary Table 1. UN targets trajectory for British Columbia (all individuals aged ≥18 months), by calendar year, from 2000 to 2013.** Prevalence: Adjusted PHAC estimates; Prevalence_1_: Public Health Agency of Canada (PHAC) prevalence estimates; Prevalence_2_: 2% Decrease in PHAC prevalence estimates; Prevalence_3_: 5% Decrease in PHAC prevalence estimates; Suppressed_1_: percent of all viral loads <200 copies/mL; Suppressed_2_: last viral load <200 copies/mL.

| Year | Prevalence | Prevalence_1_ | Prevalence_2_ | Prevalence_3_ | Diagnosed | On ART | Suppressed_1_ | Suppressed_2_ |
| --- | --- | --- | --- | --- | --- | --- | --- | --- |
| 2000 | 9150 | 9150 | 8967 | 8693 | 5874 | 2773 | 1280 | 1866 |
| 2001 | 9532 | 9408 | 9220 | 8938 | 6237 | 2723 | 1424 | 1938 |
| 2002 | 9783 | 9690 | 9496 | 9206 | 6527 | 2766 | 1478 | 2016 |
| 2003 | 10008 | 9936 | 9737 | 9439 | 6799 | 2843 | 1565 | 2138 |
| 2004 | 10259 | 10216 | 10012 | 9705 | 7096 | 3093 | 1871 | 2524 |
| 2005 | 10417 | 10398 | 10190 | 9878 | 7340 | 3418 | 2235 | 2888 |
| 2006 | 10447 | 10566 | 10355 | 10038 | 7486 | 3736 | 2638 | 3238 |
| 2007 | 10510 | 10790 | 10574 | 10251 | 7664 | 4139 | 2996 | 3702 |
| 2008 | 10570 | 11040 | 10819 | 10488 | 7846 | 4588 | 3400 | 4177 |
| 2009 | 10648 | 11280 | 11054 | 10716 | 8037 | 5094 | 3944 | 4672 |
| 2010 | 10688 | 11500 | 11270 | 10925 | 8203 | 5468 | 4353 | 5070 |
| 2011 | 10713 | 11700 | 11466 | 11115 | 8364 | 5920 | 4780 | 5526 |
| 2012 | 10666 | 11667 | 11433 | 11083 | 8468 | 6252 | 5156 | 5843 |
| 2013 | 10666 | 11633 | 11401 | 11052 | 8602 | 6531 | 5411 | 6156 |

**Supplementary Table 2. Diagnostic statistics and model coefficients for the generalized additive model for each outcome among all 12976 individuals (aged ≥18 months).** Prevalence: Adjusted PHAC estimates; Prevalence_1_: Public Health Agency of Canada (PHAC) prevalence estimates; Prevalence_2_: 2% Decrease in PHAC prevalence estimates; Prevalence_3_: 5% Decrease in PHAC prevalence estimates; 10% increase in the original prevalence estimate; Suppressed_1_: percent of all viral loads <200 copies/mL; Suppressed_2_: last viral load <200 copies/mL. The independent variable used in these models was calendar year.

1. **Negative binomial distribution and log link function**

| **Coefficients/Diagnostic Statistics** | Prevalence | Prevalence_1_ | Prevalence_2_ | Prevalence_3_ |
| --- | --- | --- | --- | --- |
| **Cubic B-Spines** |  |  |  |  |
| Knots | 5 | 7 | 6 | 6 |
|  |  |  |  |  |
| **Model Coefficient** |  |  |  |  |
| Significance of spline term for calendar year (p-value) | <0.0001 | <0.0001 | <0.0001 | <0.0001 |
|  |  |  |  |  |
| **Goodness of fit statistics** |  |  |  |  |
| R^2^ | 99.4% | 99.6% | 99.7% | 99.7% |
| Deviance explained | 99.6% | 99.7% | 99.8% | 99.8% |
| k-index p-value (i.e., testing appropriateness of the number of knots in the model) | 0.29 | 0.06 | 0.10 | 0.08 |
| Sum of deviance residuals | -0.01443 | -0.01573 | -0.02091 | -0.02112 |

1. **Beta distribution and the cloglog link function**

| **Coefficients/Diagnostic Statistics** | %Diagnosed/Prevalence | %Diagnosed/Prevalence1 | %Diagnosed/Prevalence2 | %Diagnosed/Prevalence3 | %On ART/Diagnosed | %Suppressed1/ On ART | %Suppressed2/ On ART | %Suppressed1/ Prevalence | %Suppressed2/Prevalence |
| --- | --- | --- | --- | --- | --- | --- | --- | --- | --- |
| **Cubic B-Splines** |  |  |  |  |  |  |  |  |  |
| Knots | 4 | 6 | 5 | 5 | 4 | 5 | 5 | 5 | 5 |
| **Model Parameters** |  |  |  |  |  |  |  |  |  |
| Significance of spline term for calendar year (p-value) | <0.0001 | <0.0001 | <0.0001 | <0.0001 | <0.0001 | <0.0001 | <0.0001 | <0.0001 | <0.0001 |
| **Goodness of fit statistics** |  |  |  |  |  |  |  |  |  |
| R^2^ | 99.9% | 99.1% | 99.1% | 99.1% | 99.6% | 99.2% | 99.4% | 99.9% | 99.8% |
| Deviance explained | 99.9% | 99.4% | 99.4% | 99.4% | 99.7% | 99.4% | 99.6% | 99.9% | 99.9% |
| k-index p-value (i.e., testing appropriateness of the number of knots in the model) | 0.10 | 0.60 | 0.59 | 0.54 | 0.11 | 0.52 | 0.89 | 0.47 | 0.50 |
| Sum of deviance residuals | 0.00430 | -0.01035 | -0.01098 | -0.01210 | -0.02808 | -0.03333 | -0.04868 | -0.01665 | -0.01631 |

**Supplementary Table 3. UN targets trajectory for British Columbia (all individuals aged ≥18 months), by calendar year, from 2000 to 2030.** Prevalence: Adjusted PHAC estimates; Suppressed_1_: having an undetectable viral load (<200 copies/mL) at all tests; Suppressed_2_: last viral load <200 copies/mL.

| Year | %Diagnosed  /Prevalence | *Standard Error* | %On ART  /Diagnosed | *Standard Error* | %Suppressed_1_  /On ART | *Standard Error* | %Suppressed_1_  /Prevalence | *Standard Error* | %Suppressed_2_  /On ART | *Standard Error* | %Suppressed_2_  /Prevalence | *Standard Error* |
| --- | --- | --- | --- | --- | --- | --- | --- | --- | --- | --- | --- | --- |
| 2000 | 65% |  | 47% |  | 46% |  | 14% |  | 67% |  | 20% |  |
| 2001 | 66% |  | 44% |  | 52% |  | 15% |  | 71% |  | 20% |  |
| 2002 | 68% |  | 42% |  | 53% |  | 15% |  | 73% |  | 21% |  |
| 2003 | 69% |  | 42% |  | 55% |  | 16% |  | 75% |  | 22% |  |
| 2004 | 70% |  | 43% |  | 61% |  | 18% |  | 82% |  | 25% |  |
| 2005 | 72% |  | 46% |  | 65% |  | 22% |  | 85% |  | 28% |  |
| 2006 | 73% |  | 50% |  | 71% |  | 26% |  | 87% |  | 31% |  |
| 2007 | 74% |  | 54% |  | 72% |  | 29% |  | 89% |  | 36% |  |
| 2008 | 75% |  | 58% |  | 74% |  | 32% |  | 91% |  | 40% |  |
| 2009 | 77% |  | 63% |  | 77% |  | 37% |  | 92% |  | 44% |  |
| 2010 | 78% |  | 66% |  | 79% |  | 41% |  | 93% |  | 48% |  |
| 2011 | 79% |  | 70% |  | 81% |  | 45% |  | 93% |  | 52% |  |
| 2012 | 81% |  | 74% |  | 82% |  | 49% |  | 93% |  | 55% |  |
| 2013 | 82% |  | 76% |  | 83% |  | 51% |  | 94% |  | 58% |  |
| 2014 | 83% | 0.01% | 78% | 0.68% | 84% | 1.02% | 55% | 0.75% | 94% | 0.51% | 62% | 0.57% |
| 2015 | 84% | 0.02% | 81% | 0.85% | 85% | 1.32% | 58% | 1.06% | 95% | 0.66% | 65% | 0.79% |
| 2016 | 85% | 0.02% | 83% | 1.00% | 86% | 1.61% | 62% | 1.37% | 95% | 0.79% | 69% | 1.00% |
| 2017 | 87% | 0.02% | 85% | 1.13% | 87% | 1.87% | 66% | 1.68% | 96% | 0.91% | 72% | 1.20% |
| 2018 | 88% | 0.03% | 87% | 1.22% | 88% | 2.11% | 69% | 1.97% | 96% | 1.01% | 75% | 1.39% |
| 2019 | 89% | 0.03% | 89% | 1.28% | 89% | 2.32% | 72% | 2.23% | 96% | 1.10% | 78% | 1.54% |
| 2020 | 90% | 0.03% | 91% | 1.30% | 90% | 2.49% | 76% | 2.45% | 97% | 1.17% | 81% | 1.65% |
| 2021 | 91% | 0.03% | 92% | 1.29% | 91% | 2.64% | 79% | 2.62% | 97% | 1.23% | 84% | 1.73% |
| 2022 | 91% | 0.03% | 94% | 1.25% | 92% | 2.76% | 82% | 2.73% | 97% | 1.27% | 86% | 1.76% |
| 2023 | 92% | 0.03% | 95% | 1.19% | 93% | 2.84% | 85% | 2.77% | 97% | 1.30% | 89% | 1.75% |
| 2024 | 93% | 0.03% | 96% | 1.10% | 93% | 2.89% | 88% | 2.74% | 98% | 1.32% | 91% | 1.69% |
| 2025 | 94% | 0.03% | 97% | 0.99% | 94% | 2.92% | 90% | 2.64% | 98% | 1.33% | 93% | 1.59% |
| 2026 | 94% | 0.03% | 98% | 0.87% | 95% | 2.91% | 92% | 2.48% | 98% | 1.32% | 94% | 1.46% |
| 2027 | 95% | 0.03% | 98% | 0.75% | 95% | 2.89% | 94% | 2.27% | 98% | 1.31% | 96% | 1.30% |
| 2028 | 96% | 0.03% | 99% | 0.62% | 96% | 2.83% | 95% | 2.01% | 98% | 1.28% | 97% | 1.12% |
| 2029 | 96% | 0.03% | 99% | 0.50% | 96% | 2.76% | 97% | 1.73% | 99% | 1.25% | 98% | 0.94% |
| 2030 | 97% | 0.03% | 99% | 0.40% | 97% | 2.66% | 98% | 1.43% | 99% | 1.22% | 98% | 0.76% |

**Supplementary Table 4. UN targets trajectory for British Columbia (all individuals aged ≥18 months), by calendar year, from 2000 to 2013.** Prevalence: Adjusted PHAC estimates; Prevalence_1_: Public Health Agency of Canada (PHAC) prevalence estimates; Prevalence_2_: 2% Decrease in PHAC prevalence estimates; Prevalence_3_: 5% Decrease in PHAC prevalence estimates

| Year | Prevalence | *Standard Error* | Prevalence_1_ | *Standard Error* | Prevalence_2_ | *Standard Error* | Prevalence_3_ | *Standard Error* |
| --- | --- | --- | --- | --- | --- | --- | --- | --- |
| 2000 | 9150 |  | 9150 |  | 8967 |  | 8693 |  |
| 2001 | 9532 |  | 9408 |  | 9220 |  | 8938 |  |
| 2002 | 9783 |  | 9690 |  | 9496 |  | 9206 |  |
| 2003 | 10008 |  | 9936 |  | 9737 |  | 9439 |  |
| 2004 | 10259 |  | 10216 |  | 10012 |  | 9705 |  |
| 2005 | 10417 |  | 10398 |  | 10190 |  | 9878 |  |
| 2006 | 10447 |  | 10566 |  | 10355 |  | 10038 |  |
| 2007 | 10510 |  | 10790 |  | 10574 |  | 10251 |  |
| 2008 | 10570 |  | 11040 |  | 10819 |  | 10488 |  |
| 2009 | 10648 |  | 11280 |  | 11054 |  | 10716 |  |
| 2010 | 10688 |  | 11500 |  | 11270 |  | 10925 |  |
| 2011 | 10713 |  | 11700 |  | 11466 |  | 11115 |  |
| 2012 | 10666 |  | 11667 |  | 11433 |  | 11083 |  |
| 2013 | 10666 |  | 11633 |  | 11401 |  | 11052 |  |
| 2014 | 10673 | 122 | 11758 | 133 | 11489 | 137 | 11140 | 134 |
| 2015 | 10667 | 164 | 11800 | 184 | 11514 | 190 | 11166 | 186 |
| 2016 | 10662 | 208 | 11842 | 236 | 11539 | 245 | 11192 | 240 |
| 2017 | 10656 | 252 | 11884 | 290 | 11564 | 301 | 11217 | 295 |
| 2018 | 10650 | 296 | 11926 | 344 | 11589 | 358 | 11243 | 350 |
| 2019 | 10645 | 340 | 11968 | 399 | 11614 | 415 | 11269 | 406 |
| 2020 | 10639 | 385 | 12011 | 454 | 11639 | 472 | 11295 | 463 |
| 2021 | 10634 | 429 | 12054 | 510 | 11664 | 530 | 11321 | 519 |
| 2022 | 10628 | 474 | 12097 | 567 | 11689 | 588 | 11347 | 577 |
| 2023 | 10623 | 518 | 12140 | 624 | 11714 | 647 | 11373 | 634 |
| 2024 | 10617 | 563 | 12183 | 681 | 11739 | 706 | 11399 | 692 |
| 2025 | 10612 | 607 | 12226 | 739 | 11765 | 765 | 11425 | 750 |
| 2026 | 10606 | 652 | 12270 | 797 | 11790 | 824 | 11451 | 808 |
| 2027 | 10600 | 696 | 12314 | 855 | 11815 | 884 | 11478 | 866 |
| 2028 | 10595 | 740 | 12357 | 914 | 11841 | 944 | 11504 | 925 |
| 2029 | 10589 | 785 | 12401 | 974 | 11866 | 1004 | 11530 | 984 |
| 2030 | 10584 | 829 | 12445 | 1034 | 11892 | 1064 | 11557 | 1044 |

**Supplementary Figure 1. Methodology for Adjusted Prevalence Estimates.**

The adjusted prevalence estimates used in this study were based on the proportion undiagnosed estimated by the Public Health Agency of Canada (PHAC) and on the number diagnosed based on the STOP HIV/AIDS cohort. In this calculation, we utilized available data from PHAC from 1996 to 2011 and the most recent released data in 2014. This diagram shows the original PHAC estimates (column 1) and the adjusted estimates (column 8). The diagram below shows in detail how each number was derived. The reason why we decided to adjust the prevalence provided by PHAC was due to the fact that if we multiplied the 11600 prevalence and the percent diagnosed in 2014, we would obtain that approximately 9651 individuals were diagnosed in 2014. This represents a significant increase (10%) in only 1 year of data. Thus, instead, we interpolated the proportion diagnosed between the years 2000 and 2014 and obtained the values in column 6. Next, we applied this new proportion to calculate the adjusted prevalence from 2001 onwards as shown in column 8.


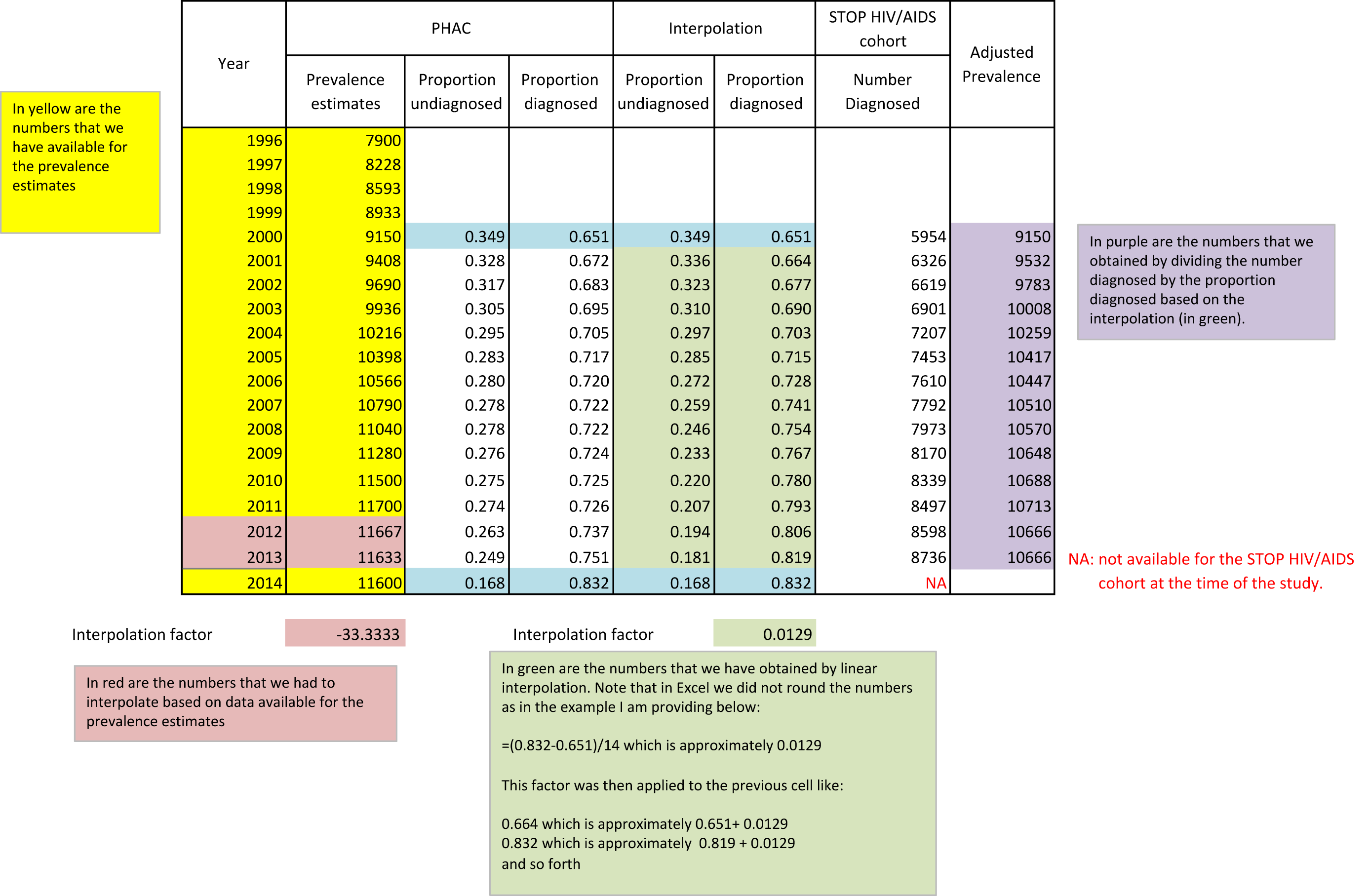

Supplement: Supplementary file 1 — Data S1. Description of the databases in the STOP HIV/AIDS cohort. Table S1. UN targets trajectory for British Columbia (all individuals aged ≥18 months), by calendar year, from 2000 to 2013. Table S2. Diagnostic statistics and model coefficients for the generalized additive model for each outcome among all 12976 individuals (aged ≥18 months). (A) Negative binomial distribution and log link function. (B) Beta distribution and the cloglog link function. Table S3. UN targets trajectory for British Columbia (all individuals aged ≥18 months), by calendar year, from 2000 to 2030. Table S4. UN targets trajectory for British Columbia (all individuals aged ≥18 months), by calendar year, from 2000 to 2013. Figure S1. Methodology for adjusted prevalence estimates. [file JIA2-20-e25011-s001.docx]
